# Redundant Role of Protein Kinase C Delta and Epsilon during Mouse Embryonic Development

**DOI:** 10.1371/journal.pone.0103686

**Published:** 2014-08-01

**Authors:** Sergio Carracedo, Frank Sacher, Gudrun Brandes, Ursula Braun, Michael Leitges

**Affiliations:** 1 The Biotechnology Centre of Oslo, University of Oslo, Oslo, Norway; 2 Max Planck Institute of Experimental Endocrinology, Hannover, Germany; 3 Zellbiologie im Zentrum Anatomie Gebäude I4, Raum, Germany; University of Regensburg, Germany

## Abstract

Protein Kinase C delta and epsilon are mediators of important cellular events, such as cell proliferation, migration or apoptosis. The formation of blood vessels, i.e., vasculo- and angiogenesis, is a process where these isoforms have also been shown to participate. However, mice deficient in either Protein Kinase C delta or epsilon are viable and therefore their individual contribution to the formation of the vasculature appeared so far dispensable. In this study, we show that double null mutation of Protein Kinase C delta and epsilon causes embryonic lethality at approximately E9.5. At this stage, whole mount staining of the endothelial marker CD31 in double null embryos revealed defective blood vessel formation. Moreover, culture of double deficient mouse allantois showed impaired endothelial cell organization, and analyses of double deficient embryo sections showed dilated vessels, decreased endothelial-specific adherent junctions, and decreased contact of endothelial cells with mural cells. Protein kinase C delta and epsilon also appeared essential for vascular smooth muscle cell differentiation, since α-smooth muscle actin, a classical marker for vascular smooth muscle cells, was almost undetectable in double deficient embryonic aorta at E9.5. Subsequent qPCR analyses showed decreased *VE-cadherin*, *Vegfr2*, *Cd31*, *Cdh2*, *Ets1*, and *Fli-1*, among other angiogenesis related transcripts in double deficient embryos. Taken together, these data suggest for the first time an *in vivo* redundant role between members of the novel Protein Kinase C subfamily that allows for mutual compensation during mouse embryonic development, with vasculogenesis/angiogenesis as an obvious common function of these two Protein Kinase Cs. Protein Kinase C delta and epsilon might therefore be useful targets for inhibiting vasculo- and/or angiogenesis.

## Introduction

The protein kinase C (PRKC) family consists of ten serine/threonine kinases grouped into three subfamilies according to their dependence on their biochemical properties and sequence homologies: classical PRKCs (cPRKCs, α, β_I_, β_II_, and γ), dependent on diacylglycerol (DAG) and Ca^2+^; novel PRKCs (nPRKCs, δ, ε, η and θ), dependent on DAG but not Ca^2+^; and atypical PRKCs (aPRKCs, ζ, ι/λ), DAG and Ca^2+^ independent. PRKC delta and epsilon (PRKCD and PRKCE) have generally different or even opposite roles [1], but both are important in several pathological scenarios, such as diabetic retinopathy [2] and fibrosis [3], respectively. However, their *in vivo* role in the formation of blood vessels is not completely understood. Previous studies on endothelial PRKCD and PRKCE have suggested roles for nPRKCs in the formation of blood vessels, such as sprouting angiogenesis [4], [5], endothelial lumen formation [6], basal barrier function [7], as well as cell migration [8], [9] and proliferation [5], [10] in different species. Regarding endothelial cell signalling, PRKCE is suggested to induce FGF-2 exocytosis (and in turn endothelial cell proliferation and sprouting [5]), as well as VEGFR2 expression and activation, thus affecting downstream targets via Akt [11]. Activation of PRKCD by VEGF via PI3K regulates vasculogenesis in embryonic stem cells [4]. PRKCD is also important in blood vessel formation under pathological conditions, such as retinopathy [2] or schemic limbs [12] in diabetic mice. However, PRKCD activation prevents instead of promoting vessel formation in diabetes.

The formation of blood vessels can be a *de novo* synthesis, which is known as vasculogenesis, or instead derive from pre-existing vessels, which is known as angiogenesis. During embryonic vasculogenesis, endothelial cells develop from both angioblasts present within the blood islands formed in the extraembryonic mesoderm of the yolk sac and angioblasts differentiated from mesodermal progenitors within the embryo [13], [14]. During angiogenesis, the formation of blood vessels can occur via different mechanisms, such as intussusception, where a pre-existing vessel splits into new vessels by the insertion of columns of tissue, or sprouting angiogenesis, where a new vessel will branch out from a pre-existing one. Different signaling pathways, such as FGF, VEGF and TGFβ are involved in the formation of blood vessels [14]. Consistently with roles for VEGF and FGF in angiogenesis, ERK signalling appears essential in this scenario during mouse embryogenesis, since its deficiency leads to lethality due to an angiogenic phenotype [15]. Among transcription factors, ETS1 and FLI-1 are also important in angiogenesis [16], [17]. In this study, we show for the first time that PRKCD and PRKCE have an *in vivo* redundant role in the formation of blood vessels during mouse embryogenesis involving endothelial proliferation and vascular smooth muscle cell (VSMC) differentiation. Our data define PRKCD and PRKCE as potential targets for inhibiting vasculo- and/or angiogenesis.

## Materials and Methods

### Animals and embryo collection

*Prkcd* and *Prkce* single mutant mice have been previously described [18], [19]. Generation of mice carrying the mutated allele for both *Prkcd* and *Prkce* was achieved by intercrossing double heterozygous mice for the corresponding isoforms. All animal work conducted for this study was approved by The Norwegian Institute of public health (Nasjonalt folkehelseinstituttet) and performed according to Norwegian legislation. The different pregnancy stages were established upon observation of vaginal plug at midday, which was considered as E0.5.

### Immunohistochemistry and immunocytochemistry

4 µm sections were serially dewaxed 3×10 min in Histochoice clearing agent (Sigma), 2×5 min in 100% ethanol, and 3 min in 90%, 70% and 50% ethanol. Sections were then immersed in water for at least 5 min and boiled in citrate buffer (pH 6.0) for 2 min. Next, sections were rinsed in PBS and blocked overnight with 30% FCS in PBS 0.1% Triton X-100. Sections were then incubated with the corresponding primary antibodies in PBS 10% FCS: Rabbit anti mouse CD31 (1∶100, Abcam), rabbit anti mouse VE-cadherin (1∶800, Abcam) or monoclonal Cy3-conjugated antimouse α-SMA (1∶1000, Sigma). CD31 was detected using the DAB method (Biogenex), whereas VE-cadherin was incubated in cy3-conjugated goat anti rabbit (1∶500, Life Technologies). For immunocytochemistry, MEECs and MEFs were cultured for two days in gelatin coated coverslips, fixed for 15 min in 4% PFA, permeabilized for 10 min with PBS 0.25% Triton X-100, blocked in PBS 10% FCS and 0.05% Tween 20 (PBST) for one hour at room temperature, incubated with PBST containing rabbit antimouse CD31 (1∶50, Abcam) overnight at 4°C, washed 3×10 min in PBST, and incubated with cy3-conjugated goat anti-rabbit (1∶500, Life Technologies) for two hours at room temperature. Cells were photographed upon mounting with Dapi containing medium (Life Technologies).

### Whole mount immunostaining

E9.5 embryos were fixed overnight at 4°C in a methanol-DMSO mix (4∶1), bleached next day in a methanol-DMSO-30% H_2_O_2_ mix (4∶1∶1) for 2 h at 4°C, washed three times in methanol, rehydrated in PBS containing decreasing methanol concentrations (75–50–25%), washed twice in PBS, digested for 5 min in PBS 0.1% Triton X-100 containing 20 µg/ml protease K, washed two times in PBS 0.1% Triton X-100, incubated in 0.1% GDA in PBS 0.1% Triton X-100 for 15 min, washed in PBS 0.1% Triton X-100, incubated in 2 mg/ml glycin in PBS, incubated with blocking buffer (2% skimmed milk in PBS 0.1% Triton X-100) for 2 h at room temperature, incubated overnight at 4°C with rat antimouse CD31 (BD Pharmingen) or α-SMA (Sigma) in blocking buffer at a dilution of 1∶200 and 1∶500 respectively, washed next day 5 times for one hour each time in blocking buffer, incubated in peroxidase-conjugated goat antirat and antimouse IgGs, respectively (1∶200, Jackson Immunoresearch), and developed using the DAB method according to the manufacturer's instructions (Biogenex). Embryos were postfixed in 0.1%GDA in PBS, passed into increasing concentrations of methanol in PBS (25, 50 and 75%) and stored in 100% methanol until photographed.

### LacZ staining

LacZ staining of embryos was performed as previously described [20], [21], except that 2.5% GDA in PBS was used for fixing embryos upon collection. For section staining with eosin, 4 µm thick LacZ stained sections were further immersed in eosin for 2 min, rinsed in runnig water for 5 min, dehydrated serially 3×2 min in 90% ethanol, 3×2 min in 100% ethanol, 2 min in 1∶1 solution of ethanol: histochoice clearing agent (Sigma), 3×2 min in histochoice clearing agent, then mounted with Entellan (Merck), and photographed. Pictures of whole embryos were acquired using a Zeiss stereoscope equipped with camera and Axiovision software, whereas a Nikon Eclipse Ti microscope was used to photograph LacZ/eosin stained embryo sections.

### Allantois assay

Allantoises were prepared, cultured, photographed and quantified as previously described [22], [23], except that gelatin was used to coat wells prior culturing allantoises, and rat anti mouse CD31 (Pharmingen) was used as an endothelial marker to immunostain blood vessels. A Nikon Eclipse Ti microscope was used to acquire photographs.

### Electron microscopy

Embryos were fixed in 2.5% GDA in 0.1 M sodium cacodylate, pH 7.3. After postfixation with 2% osmium tetroxide dissolved in 0.1 M sodium cacodylate and dehydration in graded alcohols, embryos were embedded in Epon. Sections stained with uranyl acetate and lead citrate were examined in a Philips EM 301 electron microscope.

### Hematoxylin & Eosin staining

Sections were dewaxed as above described and subsequently submerged in Hematoxylin solution for 5 min. Upon rinsing with running water for 5 min, sections were immersed in eosin solution for 2 min and rinsed for 5 in running water. Next, sections were serially dehydrated by being immersed for 2 min in each of the following solutions: 3×95% ethanol, 3×100% ethanol, 1×50∶50 ethanol:xylol (Invitrogen), and 2×100% xylol. Sections were then mounted with Entellan (Merck) and photographed.

### Cell culture

WT and *Prkcd*−/− MEECs were generated and cultured as previously described [20]. Their purity was assessed by detection of CD31 (described above in the immunocytochemistry method). MEFs used as negative control for immunodetection of CD31 were isolated as previously described [24].

### Western Blotting

Cells were lysed on a solution containing Tris/HCl 50 mM, EDTA 2 mM, EGTA 10 mM, protease inhibitor cocktail (Sigma) and 0.3% beta-mercaptoethanol (Biorad), and centrifuged at 300 rfc to collect the cytosolic fraction, upon which SDS-PAGE was performed. After protein transfer, nitrocellulose membranes (GE Healthcare) were blocked with 3% non-fat dry milk (Marvel) in PBS 0.1% Tween 20 (TBS-T) for one hour. Next, membranes were incubated with rabbit anti mouse PRKCD (Santa Cruz Biotechnologies) and rabbit anti GAPDH (Cell Signalling Technology) were used overnight at 4°C at a concentration of 1∶1000 and 1∶5000, respectively, and incubation of membranes in goat anti-rabbit horseradish peroxidase-conjugated secondary IgGs (Jackson Immunoresearch, 1∶5000) was done for two hours at room temperature. Upon washing with PBS 0.1% Tween 20, membranes were developed using SuperSignal West Pico kit (Thermo-Scientific Pierce) and X-ray films (GE-Healthcare), which were further scanned to create the corresponding figure.

### Quantitative Polymerase Chain Reaction (qPCR)

Total mRNA from embryos was isolated by using the RNeasy kit (Qiagen) according to the manufacturer's instructions. Sixty ng of total RNA was used to generate total cDNA using the iScript cDNA synthesis kit (Biorad). Subsequently, 1 µl cDNA was used to amplify the pertinent genes via qPCR using iCycler IQ Real-Time PCR Detection System and iQ SYBR green master mix (Biorad) according to the manufacturer's instructions. Absolute mean quantifications of triplicates for each gene and genotype were normalized to those corresponding to GAPDH, and fold changes in mutant embryos were calculated by calibrating their GAPDH normalized values to those obtained in wild type embryos. Bars for each gene plotted in the graph represent mRNA fold change of normalized double deficient versus wild type values obtained in three different experiments. cDNA from independent embryos was used in each experiment. Error bars represent standard errors for normalized values from three different experiments. Two-tailed t-test was used to determine significant difference between wild type and mutant embryo values. Primer sequences for each gene were obtained from http://mouseprimerdepot.nci.nih.gov/, and are provided as Table S1.

## Results

### Embryonic lethality in *Prkcd* and *Prkce* double deficient embryos at approximately E9.5

**Figure 1:**
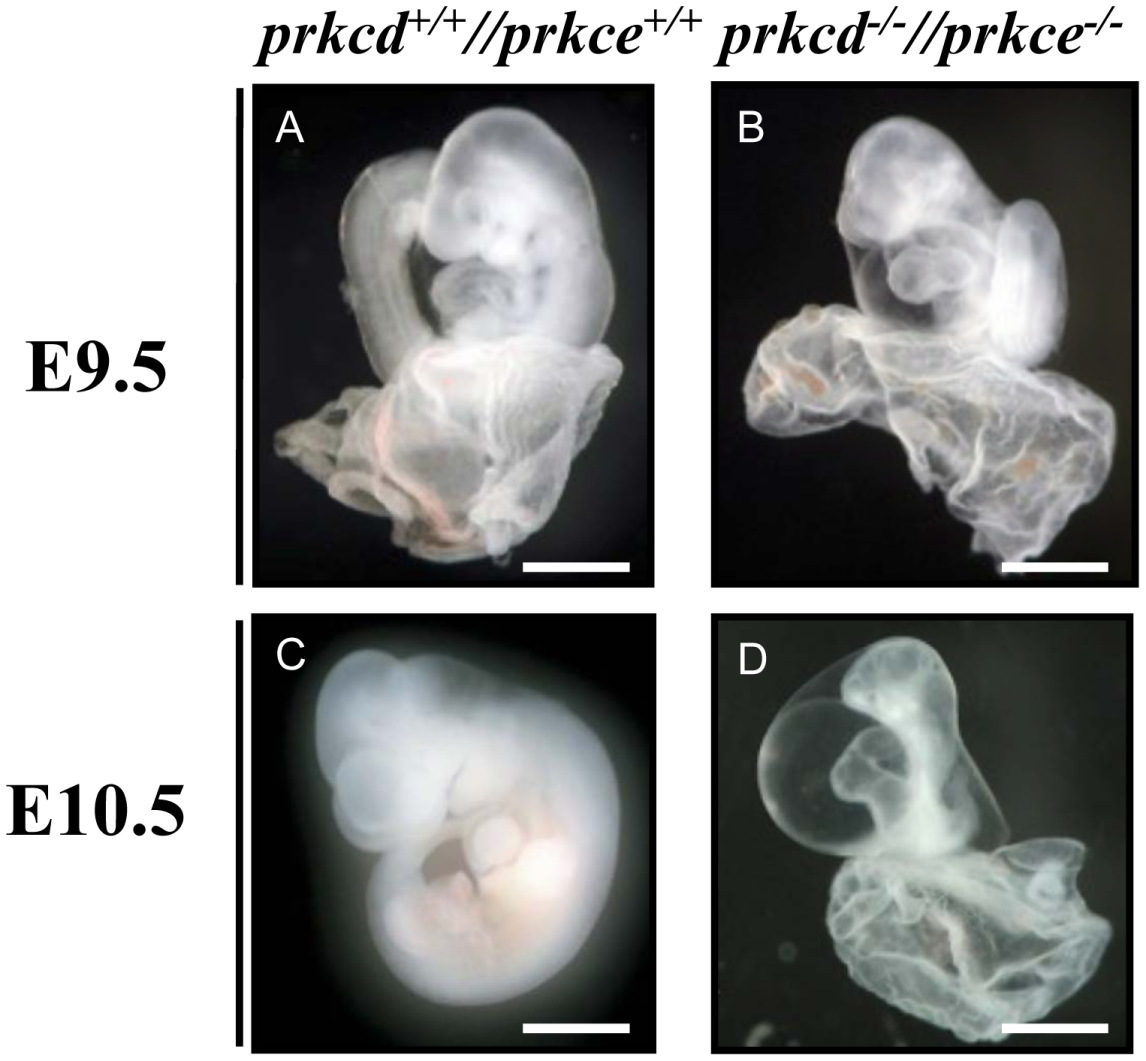
Embryonic lethality in *Prkcd* and *Prkce* double deficient mouse embryos at approximately E9.5. A and B, embryonic stage E9.5. Double null embryos (B) display growth retardation, and swollen pericardium as main phenotypes. C and D, embryonic stage E10.5, when such phenotypes became more remarkable. Scale bars  = 800 µm.

**Figure 2:**
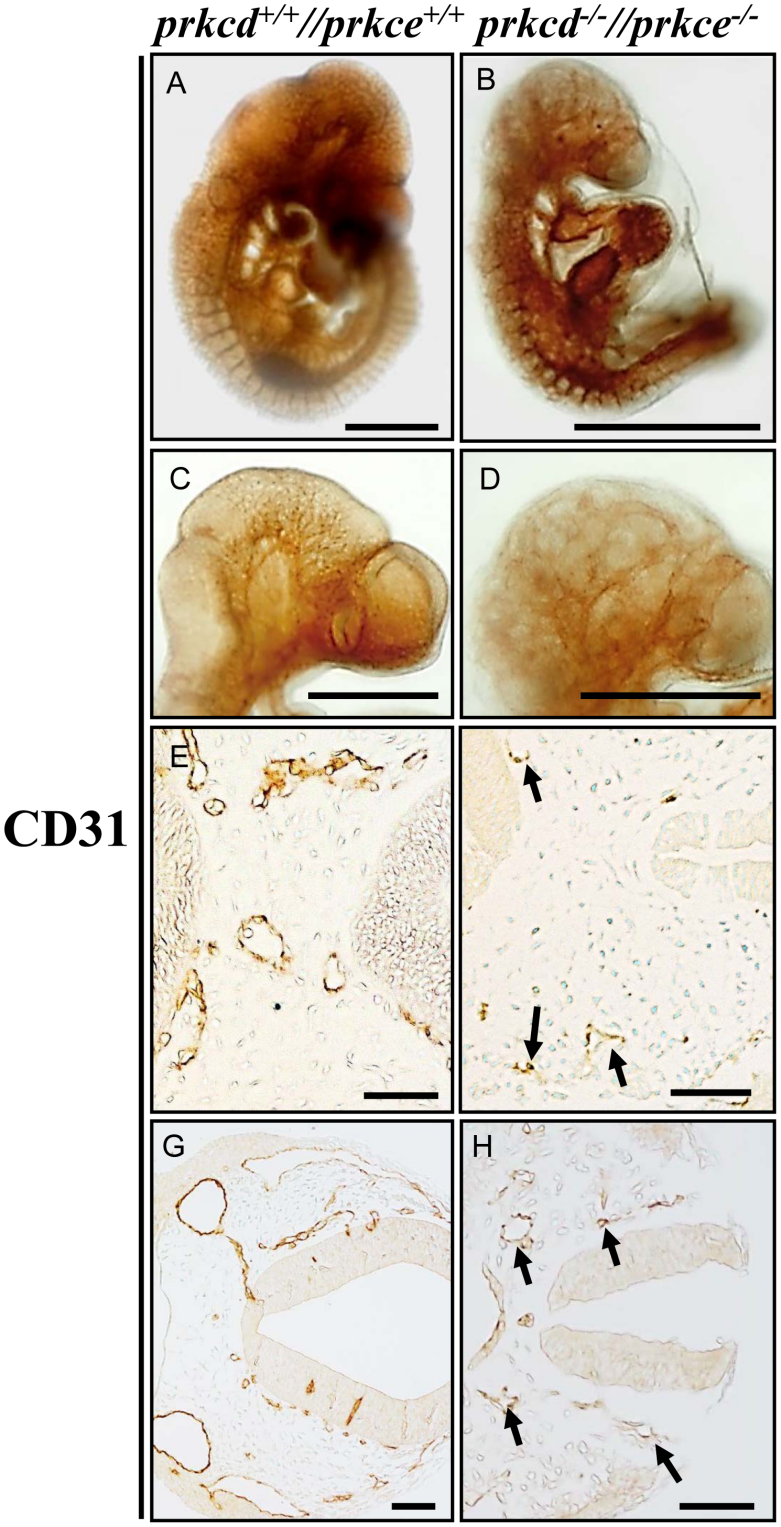
Impaired vessel formation in *Prkcd* and *Prkce* double deficient mouse embryos. Unlike in wild type counterparts (A), obvious absence of defined vascular network in double deficient embryos (B) could be observed at E9.5 by performing whole mount immunostaining of the endothelial marker CD31 (PECAM). C and D, zoom-in of PECAM immunostained wild type and double deficient embryonic heads, respectively. E–H, immunodetection via CD31 of blood vessels in head (E and F) and trunk (G and H) in E9.5 mouse embryonic transversal sections. Wild type endothelial tubes are readily detected (E and G), unlike double deficient counterparts (F and H), which are less abundant and show weaker positive staining (arrows). Scale bars  = 1000 µm (A and B), 500 µm (C and D), and 50 µm (E–H).

We have recently shown that *Prkcd* and *Prkce* have partly overlapping expression patterns during embryogenesis [20], [21]. Since the individual knockout mutants of these two genes do not display obvious embryonic phenotypes [18], [19] we established a double deficient line in order to address potential *in vivo* redundancy. Interestingly, whereas wild type, *Prkcd−/−, Prkce−/−*, *Prkcd+/−//Prkce+/−*, *Prkcd−/−//Prkce+/−* and *Prkcd+/−//Prkce−/−* embryos developed normally, no double homozygous *Prkcd−/−//Prkce−/−* offspring was observed, thus indicating an embryonic lethal phenotype (out of 150 newborns, 6% wild type, 6% *Prkcd−/−,* 5% *Prkce−/−*, 37% *Prkcd+/−//Prkce+/−*, 16% *Prkce+/−*, *Prkcd+/−*, 7% *Prkcd−/−//Prkce+/−* and 5% *Prkcd+/−//Prkce−/−* were identified). A subsequent analysis of various embryonic stages from double heterozygous crosses revealed that *Prkcd* and *Prkce* double deficiency lead to developmental defects at approximately E9.5. Unlike wild type (Fig. 1A), they displayed obvious growth retardation, mainly indicated by a reduced head size and a shortened tail, as well as swollen pericardium (Fig. 1B). Such developmental defects became more pronounced at E10.5 (Fig. 1D), whereas at later stages (e.g. E12.5) no double deficient embryos were detected due to absorption of affected embryos by the mother. To address whether the observed morphological abnormalities involved a vascular defect, we next performed whole mount and section immunostaining of wild type and *Prkcd* and *Prkce* double deficient embryos in order to compare the overall structure and distribution of the vasculature. Thus, through immunodetection of the endothelial marker CD31 in whole embryos, clearly detectable capillaries branching out from larger vessels could not be observed in the absence of *Prkcd* and *Prkce* (Figure 2B). Consistently, fewer vessels were detected in the corresponding sections (Figures 2D and2F), as well as weaker positive immunosignal for CD31 in double mutants (Figs. 2F). Wild type embryos however showed a clearly defined vessel structure overall, as expected (Figures. 2A, 2C and 2E). Thus, considering the viability of *Prkcd* and *Prkce* single deficient mice, these data show that PRKCD and PRKCE have at least one redundant role *in vivo*, and that double deficiency of these isoforms leads to impaired formation of the vasculature.

### PRKCD and PRKCE are co-expressed in mouse endothelium

**Figure 3:**
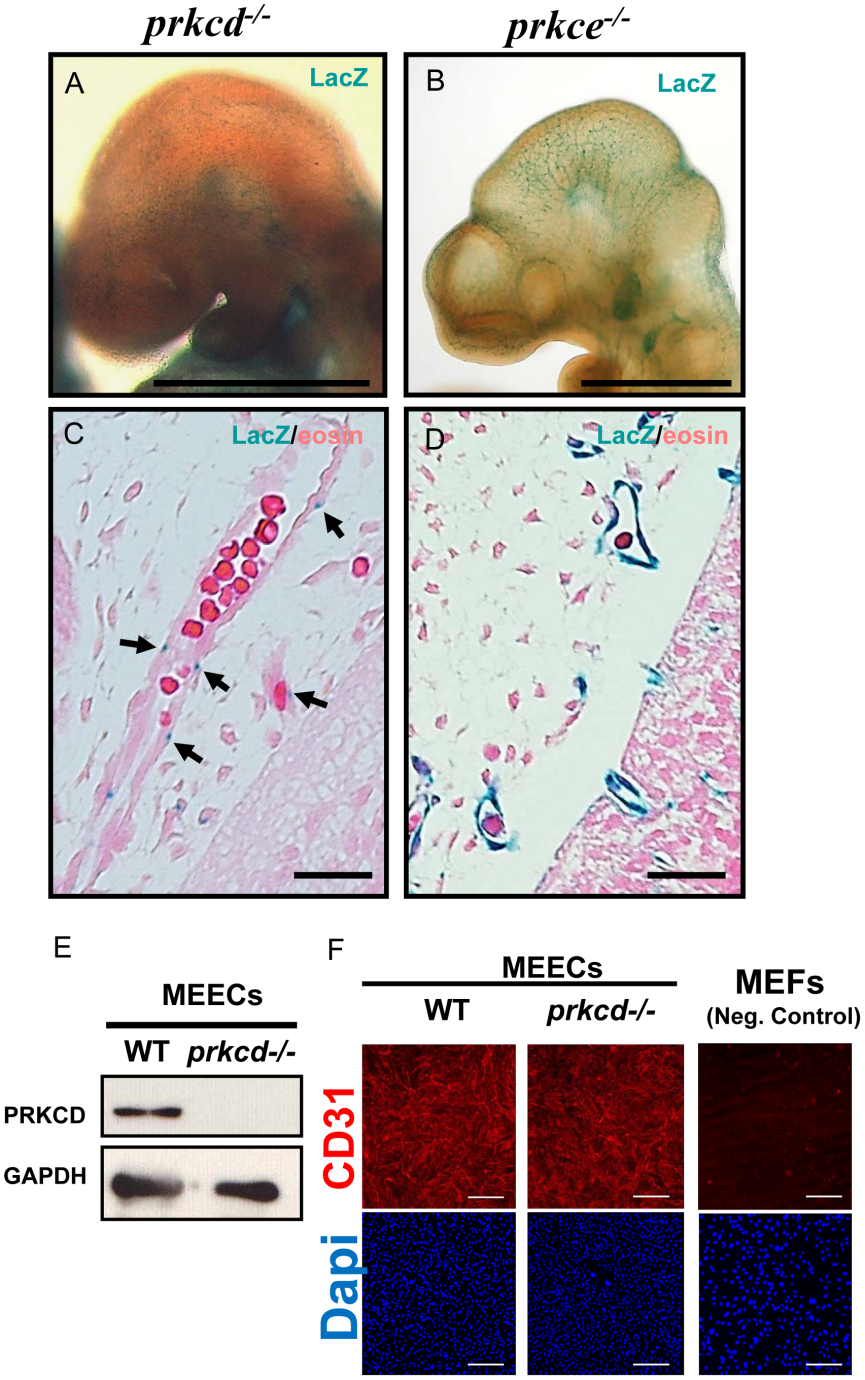
PRKCD and PRKCE are both expressed in mouse embryonic endothelium at E9.5. A, whole mount LacZ staining suggests weak expression of PRKCD in few endothelial cells. B, PRKCE appears broadly expressed in endothelium in whole mount LacZ stained embryos. C and D, LacZ and eosin stained sections confirmed the endothelial expression pattern observed for PRKCD and PRKCE in whole embryos, respectively. E, western blotting shows that wild type mouse embryonic endothelial cells (MEECs) express PRKCD. *Prkcd*−/− MEECs were used as a negative control. F, CD31 positive signal in virtually all MEECs confirms the purity of WT and *Prkcd*−/− cell populations. Mouse embryonic fibroblasts (MEFs) were used as a negative control. Expression of PRKCE in MEECs was previously demonstrated in a similar manner [20]. Scale bars  = 500 µm (A and B), 25 µm (C and D) and 200 µm (F).

Given the impaired formation of the vasculature found in our double deficient embryos (Fig. 2), we hypothesized that impaired vascular development in the absence of *Prkcd* and *Prkce* double deficiency could involve an endothelial-related defect. This would imply that both *PRKCD *and *PRKCE *would need to be expressed in endothelium. Thus, since both *Prkcd* and *Prkce* single deficient embryos contain the LacZ reporter gene under the control of the *Prkcd* and *Prkce* promoters, respectively (see material and methods), we aimed to identify the endothelium as a novel domain where PRKCD and PRKCE expressions overlap at embryonic stage E9.5 through LacZ staining (Fig. 3). Indeed, whole mount LacZ stained embryos suggested that PRKCD is expressed at discrete spots in blood vessels (Fig. 3A), whereas PKC epsilon would appear more broadly expressed in endothelium (Fig. 3B). Such observations were confirmed on the corresponding LacZ stained sections (Figs. 3C and 3D, respectively), although expression of PRKCD appeared weak compared to that of PRKCE. However, western blot analyses showed expression of PRKCD in wild type E10 mouse endothelial cells (MEECs, Fig. 3E), which suggests that PRKCD is expressed in endothelium at this embryonic stage. The purity of MEECs was characterized through fluorescent immunodetection of VE-cadherin [20] and CD31 (Fig. 3F), with mouse embryonic fibroblasts serving as a negative control. The specificity of the signal for PRKCD in wild type MEECs was confirmed by using the *Prkcd−/−* MEECs as a negative control (Fig. 3E). Thus, taken together these data suggest that PRKCD and PRKCE are co-expressed in a subset of the endothelium, and are consistent with PRKCD and PRKCE as potential redundant mediators of vascular development during mouse embryogenesis.

### Impaired cell organization in cultured *Prkcd* and *Prkce* double deficient allantois

**Figure 4:**
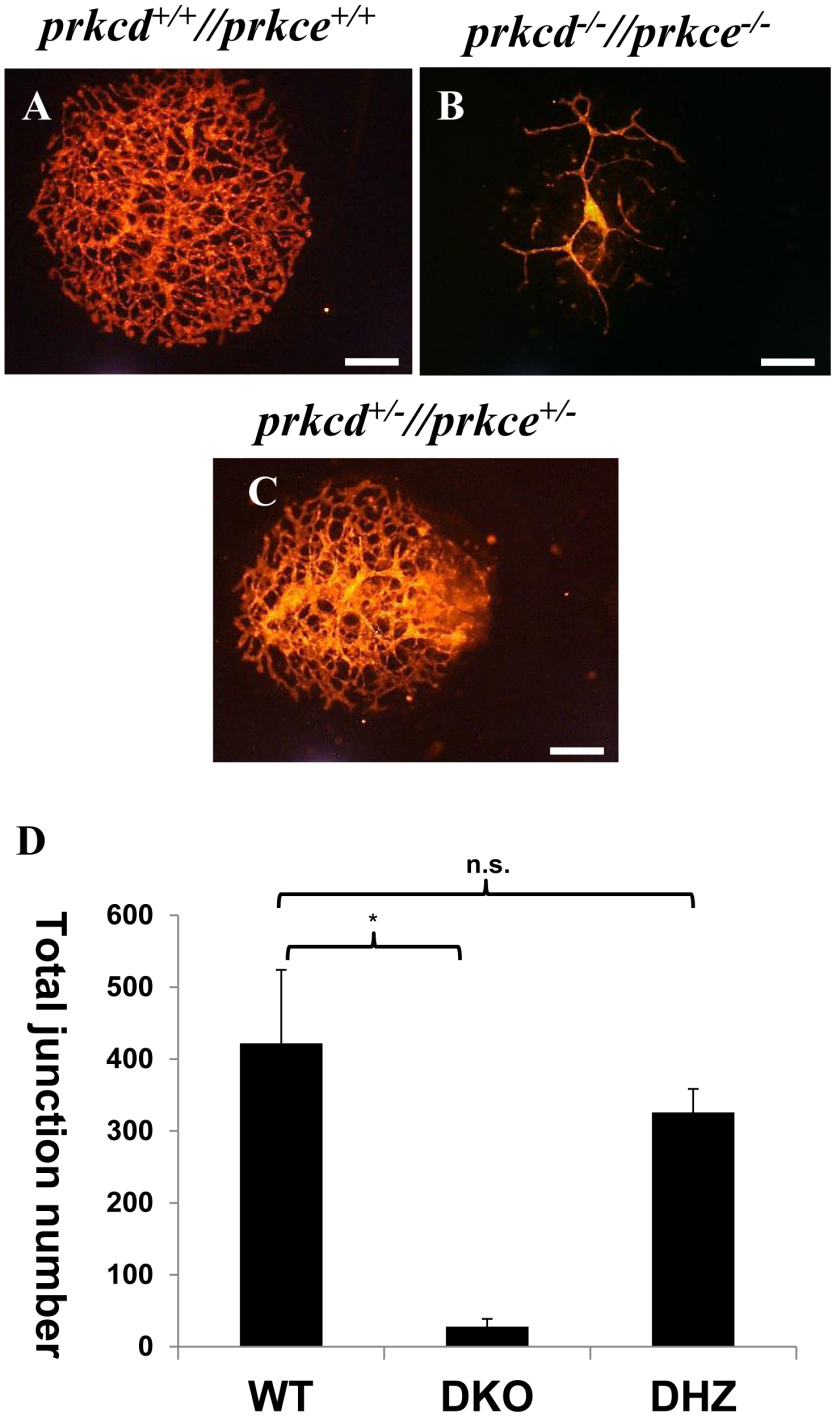
Impaired cell organization in cultured *Prkcd* and *Prkce* double deficient murine allantois. A, *in vitro* culture of wild type allantois led to the formation of a vascular plexus. B, *Prkcd* and *Prkce* double deficient allantois in culture displayed impaired formation of a vascular network. C, blood vessel formation in cultured *Prkcd* and *Prkce* double heterozygous allantois does not significantly differ from wild type counterparts. D, quantifications with the software angiotool and student t-test analyses of 3 allantoises per genotype show significantly decreased average value for total number of junctions in *Prkcd* and *Prkce* double deficient allantoises versus their wild type counterparts. Scale bars  = 500 µm. * and n.s. =  Difference between average values is (p≤0.01) and is not statistically significant, respectively.

In the mouse embryo, the allantois consists of mesodermal tissue that differentiates into the umbilical vein and artery [25], and has been used as an *in vitro* model for vascular network formation and angiogenesis [26]. Given the lack of vessel branching in our double deficient embryos, we cultured E8.5 embryonic allantois to further confirm the vascular phenotype suggested by CD31 immunostaining in whole embryos (Fig. 2). Wild type murine allantois led to the formation of a vascular plexus *in vitro* (Fig. 4A), as previously reported [25]. However, *Prkcd* and *Prkce* double deficient allantois failed to form a defined vascular network under the same conditions, as indicated by impaired formation of larger and smaller vessels (Fig. 4B). *Prkcd* and *Prkce* double heterozygosity did not significantly affect blood vessel formation in cultured allantois (Figs. 4C and D), which is consistent with the viability of the respective mice. Taken together, these data suggest that there is a key redundant role for PRKCD and PRKCE in embryonic endothelium, and therefore in the formation of embryonic vasculature.

### Impaired vessel integrity in *Prkcd* and *Prkce* double deficient embryos

**Figure 5:**
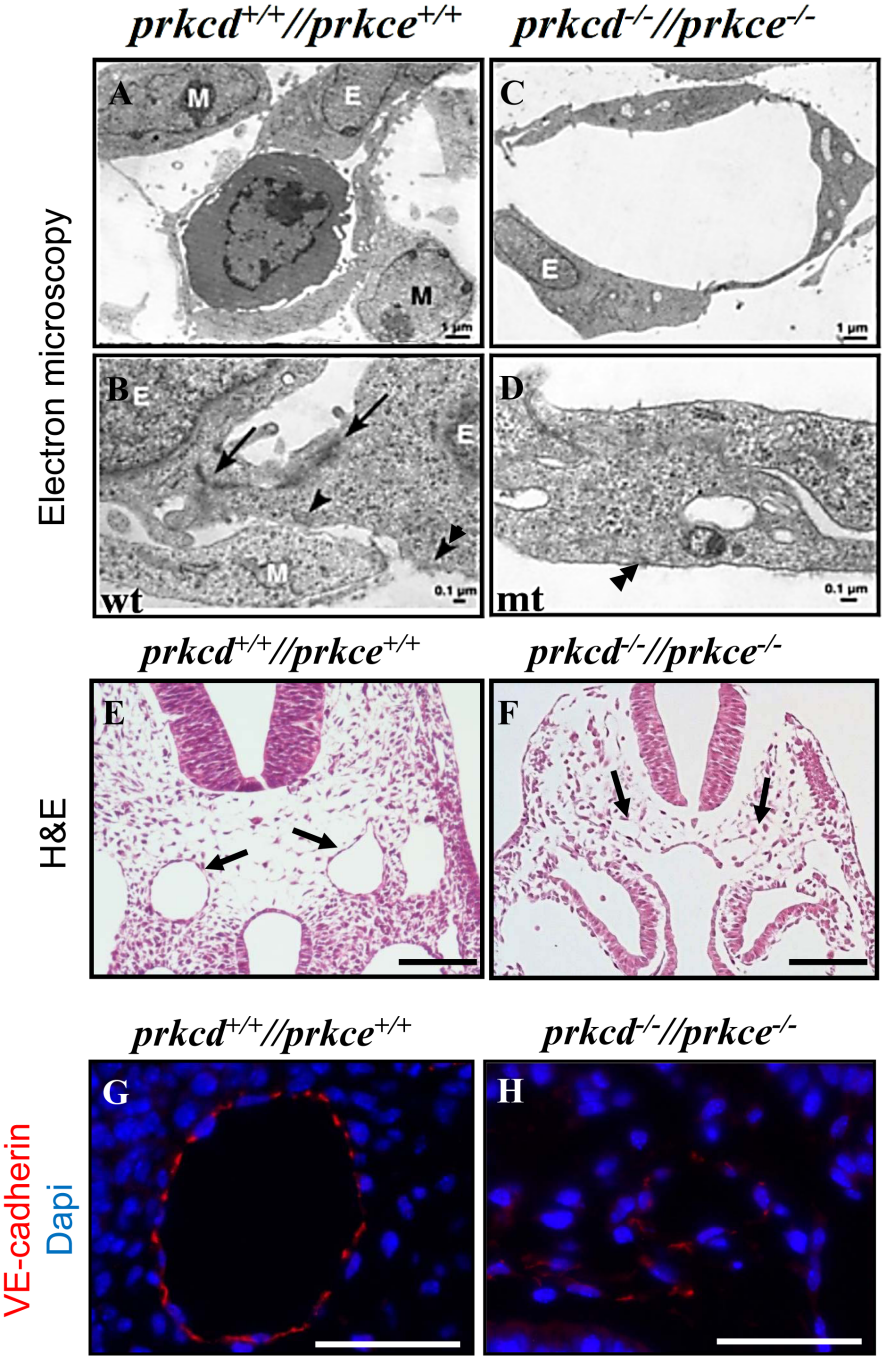
Impaired vessel structure in *Prkcd* and *Prkce* double deficient embryos. A–D, via transmission electron microscopy, wild type (A and B) sagittal sections of the E9.5 embryonic head showed endothelial cells with endothelial adherens junctions (arrows) as well as contact with surrounding mesenchymal cells (single arrowhead). However, *Prkcd* and *Prkce* double deficient sections (C and D) showed dilated vessels, and endothelial cells with decreased endothelial specific adherent junctions and decreased contact with surrounding cells. Hemidesmosomes (double arrowheads) were equally detectable in both wild type and *Prkcd* and *Prkce* double deficient embryo sections. E and F, comparison between hematoxylin & eosin stained wild type (E) versus *Prkcd* and *Prkce* double deficient (F) transversal embryo sections at E9.5 showed reduced size of dorsal aorta (arrows) in the absence of *Prkcd* and *Prkce.* G and H, immunofluorescent analyses showed that VE-cadherin is readily expressed in wild type dorsal aorta whereas its expression levels appeared reduced in double deficient sections on these embryos. E, endothelial cell; M, mesenchymal cell. Scale bars  = 1 µm (A and C), 0.1 µm (B and D), 100 µm (E and F), and 50 µm (G and H).

Since we observed impaired vessel formation in *Prkcd* and *Prkce* deficient embryos and allantois (Figs. 2 and 4, respectively), we next performed a more detailed histological analysis to obtain further information regarding blood vessel structure (Fig. 5). Via electron microscopy, wild type embryo sections showed that endothelial cells were properly interconnected and physically interacting with surrounding mural mesenchymal cells (Figs. 5A and 5B). In contrast, *Prkcd* and *Prkce* double deficient sections showed disassembled endothelial tubes with barely detectable endothelial-specific cell-cell adhesion molecules, i.e adherens junctions, as well as decreased contact with surrounding mural mesenchymal cells (Figs. 5C and 5D). Extracellular matrix-cell adhesion molecules were however detected at hemidesmosomes in both wild type and PRKCD and PRKCE double deficient embryo sections (Fig. 5B and 5D, respectively). In addition, while apparently normal in wild type sections, histochemical analyses showed a small dorsal aorta in double deficient embryo sections (Figs. 5E and 5F, respectively), which was also suggested by our findings via CD31 (Fig. 2E and 2F). Moreover, immunofluorescent detection of the VE-cadherin (CDH5), an endothelial marker expressed at endothelial adherens junctions, appeared expressed at lower levels in the absence of PRKCD and PRKCE (Fig. 5H). Taken together, these data suggest that PRKCD and PRKCE are necessary for proper assembly and development of the vasculature, and for expression of CDH5 at the endothelial cell membrane.

### Lack of differentiated vascular smooth muscle cells in *Prkcd* and *Prkce* double deficient aorta

**Figure 6:**
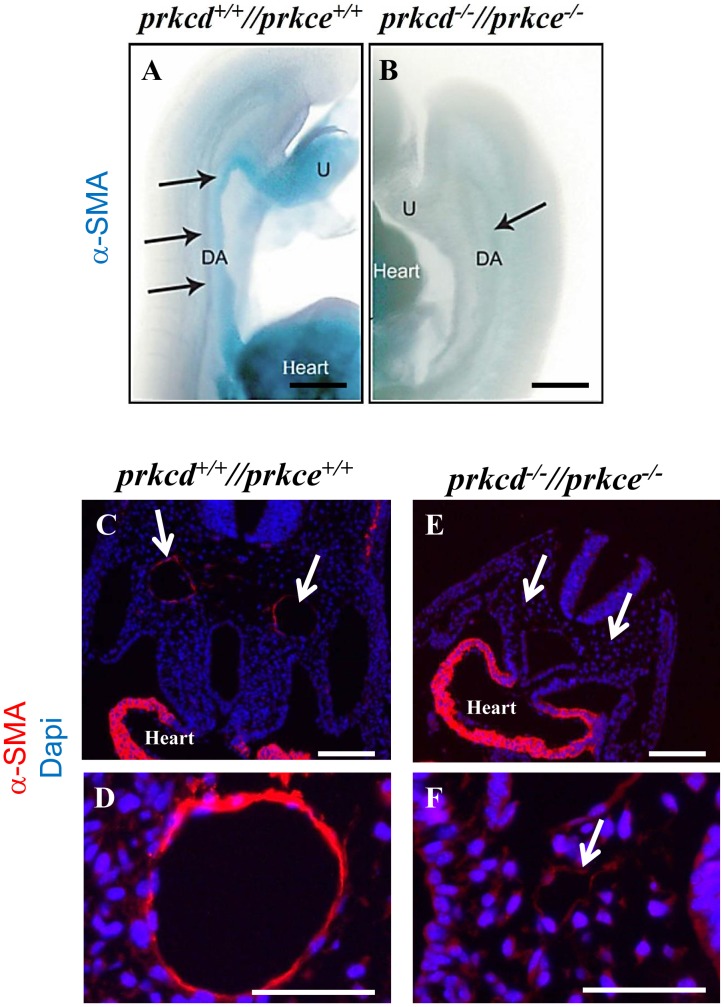
Undetectable vascular smooth muscle cell differentiation in *Prkcd* and *Prkce* double deficient dorsal aorta. A and B, immunodetection of α-SMA in whole embryos resulted in positive staining of dorsal aorta in wild type (A) but not *Prkcd* and *Prkce* double deficient (B) embryos at E9.5. C and D, wild type sections showed positive staining for α-SMA at the dorsal aorta (C and D) and heart (C) at E9.5. E and F, *Prkcd* and *Prkce* double deficient sections showed no immunosignal over the background at dorsal aorta at E9.5. Arrows indicate dorsal aorta. DA, dorsal aorta; U, umbilical cord. Scale bars  = 250 µm (A and B), 100 µm (C and E), and 50 µm (D and F).

During embryonic blood vessel formation, endothelial cells interact with surrounding mesenchymal cells and induce VSMC/pericyte differentiation [27]. Given that endothelial-mesenchymal cell interactions appeared impaired in the absence of PRKCD and PRKCE (Fig. 5), we next analysed *in vivo* mesenchymal differentiation and therefore vessel maturation in the absence of PRKCD and PRKCE. Thus, immunostaining of whole mount embryos with antibodies to alpha-smooth muscle actin (α-SMA), a marker for pericytes and VSMCs [27], allowed for detection of positive staining at dorsal aorta in wild type but not in *Prkcd* and *Prkce* double deficient embryos at E9.5 (Fig. 6A and 6B, respectively). This was further confirmed by immunofluorescent detection of α-SMA in E9.5 wild type and double deficient embryo sections (Figs. 6 C–F). Thus, these data suggest that PRKCD and PRKCE are needed for proper VSMC differentiation and therefore blood vessel stability and maturation.

### Decreased levels of endothelial markers and vasculo/angiogenic related genes in *Prkcd* and *Prkce* double deficient embryos

**Figure 7:**
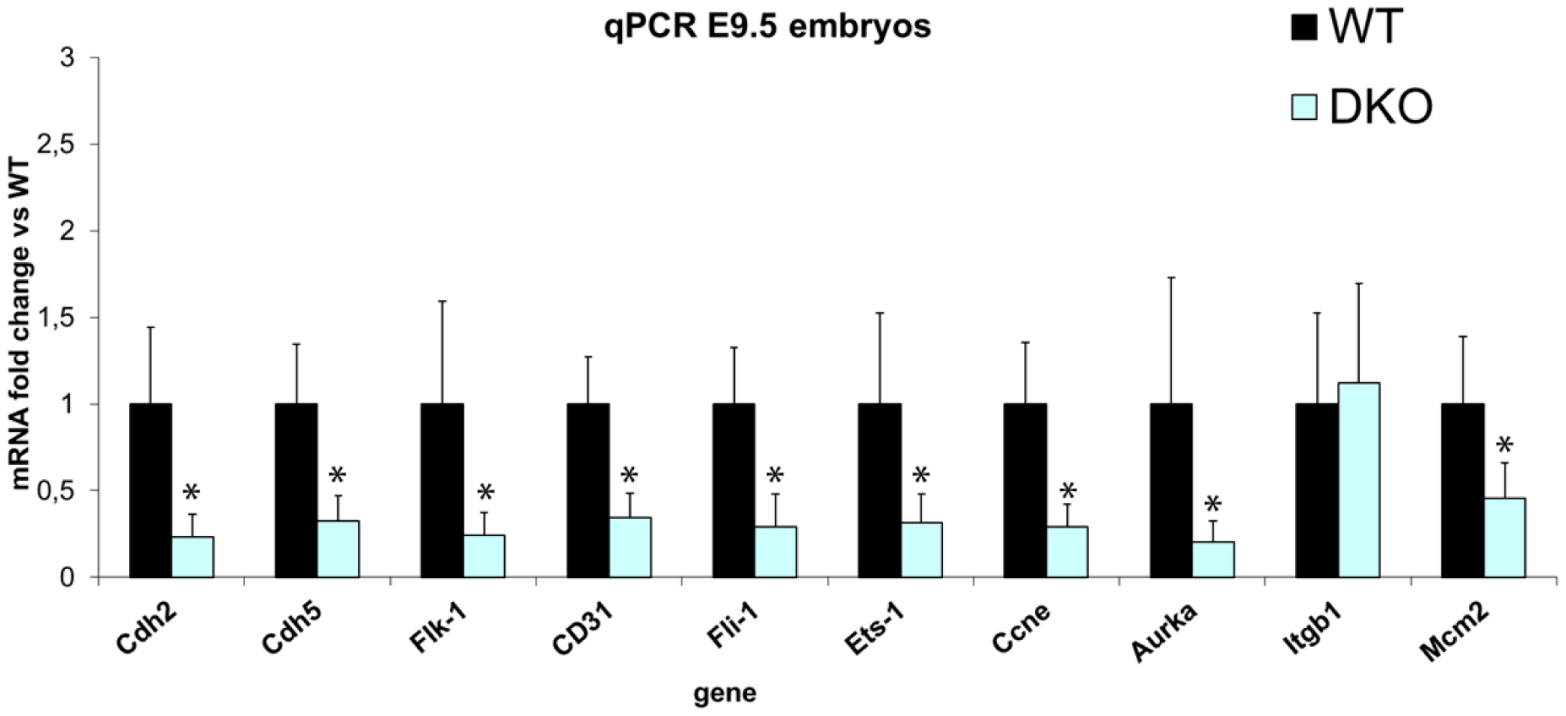
Decreased mRNA levels of vasculo and angiogenic genes in the absence of PRKCD and PRKCE. Significant downregulation of several vasculo- angiogenic genes in double *Prkcd* and *Prkce* deficient embryos suggests an important role of PRKCD and PRKCE in the formation of the vasculature. * = p<0.05.

Since our data at the protein level suggested impaired vasculature formation in the absence of PRKCD and PRKCE, we next performed a pre-screening of vasculo/angiogenic related transcripts in double deficient embryos, and found downregulation of *cdh5*, N-cadherin (*Cdh2*), and the transcription factors *Ets1* and *Fli1*, both involved in vascular development [16] (data not shown). The transcriptional regulation of these genes was confirmed via qPCR (Fig. 7). In addition, since ERK is a known downstream target for PRKCD [28] and PRKCE [11], and mouse embryos with conditional deletion of *Erk1/2* in the endothelium are not viable due to an angiogenic phenotype at approximately the same developmental stage as *Prkcd* and *Prkce* double deficient embryos, we also analyzed mRNA levels of angiogenic genes that were found most significantly regulated in embryos lacking endothelial ERK1/2 [15]. Indeed, *Flk-1*, *Ccne*, *Aurka*, and *Mcm2* all appeared downregulated in the absence of PRKCD and PRKCE. However, *Itgb1*, also significantly downregulated in the absence of endothelial ERK1/2, did not seem to be dependent on the expression of PRKCD and PRKCE. This is consistent with the detection of hemidesmosomes (which contain ITGB1) in *Prkcd* and *Prkce* double deficient embryo sections (Fig. 5). Thus, these data suggest that PRKCD and PRKCE regulate genes involved in blood vessel formation at the transcriptional level.

## Discussion

We recently showed that PRKCD and PRKCE display similar expression patterns in the mouse embryo during midgestation [20], [21]. Such similarity lead us to hypothesize that PRKCD and PRKCE could have redundant function(s) *in vivo* that would explain the viability of both *Prkcd* and *Prkce* single deficient mice [18], [19]. For this reason, a mouse line containing null mutations for both *Prkcd* and *Prkce* was generated. Given the strong expression of these isoforms within the nervous system [20], [21], finding an obvious phenotype within this domain would not have come as a surprise. However, we found that *Prkcd* and *Prkce* double deficiency caused lethality in mouse at E9.5, a stage at which the nervous system has not started to develop yet. The main observed phenotypes were growth retardation and swollen pericardium, which suggested impaired vascular development at this stage and a potential contributor to embryonic lethality (Fig. 1). This was further supported by immunodetecting impaired vascular network in double deficient embryos (Fig.2). Thus, it seems obvious that either PRKCD or PRKCE must be present in the mouse embryo for adequate development of the vasculature. An endothelial related defect implies that both PRKCD and PRKCE need to be expressed in endothelium. However, whereas we previously reported obvious expression of PRKCE in endothelium from E.12.5 and onwards via a LacZ reporter gene under the control of the *Prkce* promoter and western blotting [20], a similar in vivo expression analysis for PRKCD did not suggest clear expression of this isoform in endothelial cells during mouse midgestation [29]. Some modifications in the LacZ staining protocol (see material and methods) allowed us to obtain more prominent LacZ signal for both PRKCD and PRKCE (Fig. 3), and thus better detect indirect expression of these isoforms at E9.5. The detection of PRKCD at discrete spots throughout the endothelium may indicate that it has a significant role in a small endothelial cell subpopulation, perhaps tip cells, and therefore in endothelial migration during sprouting angiogenesis, although further studies are needed in this regard. Indeed, absence of endothelial PRKCD has been shown to delay reendothelialization *in vivo* via decreased vasohibin-1 in a mouse model of injured artery [8]. PRKCE has also been shown to take part in endothelial function, for example in a novel mechanism where FGF2, a known pro-angiogenic factor, has been linked to endothelial cell sprouting upon PRKCE activation via VEGF signaling [5]. This provides a novel mechanism that might at least partly contribute to the development of murine vasculature *in vivo*. Downstream effectors of signaling cascades involved in PRKCE-dependent formation of blood vessels may also include AKT and eNOS [11].

Our observations that *Prkcd* and *Prkce* double deficient vessels a) did not appear adequately assembled into a vascular network (Figs. 2 and 4), and b) showed decreased detectable cell-cell adhesion molecules at the cell membrane that could allow for endothelial/mesenchymal cell interaction and endothelial interconnection respectively (Fig. 5), strongly suggested impaired vessel maturation in the absence of PRKCD and PRKCE. This was further confirmed by the absence of immnunodetectable α-SMA, a marker for VSMCs and pericytes [30], in double deficient aorta (Fig. 6). Thus, these data suggest a potential role for PRKCD and PRKCE in the crosstalk between endothelial and mesenchymal cells in order to allow for recruitment of the latter, their further differentiation into VSMCs/pericytes, and hence the formation of a mature and functional vascular network.

We also wanted to address whether the apparent decrease of detectable CDH5 in embryo sections was the result of lower transcription levels of the corresponding gene. Through qPCR analysis (fig. 7) we could confirm a previous screening of transcripts (data not shown) and detect downregulated *cdh5* (Fig. 7). However, it is likely that the observed lower levels of *Cdh5* in double deficient embryos not only reflect lower expression of these molecules in endothelial cells, but also a lower endothelial cell number in double deficient embryos, given the observed underdeveloped vasculature. Nevertheless, our data via electron and fluorescent microscopy (Fig. 5) together with the fact that *Cdh5* deficient mice also display a vascular lethal phenotype [31] suggest that PRKCD and PRKCE redundantly act upstream CDH5 expression. Such redundancy may also exist for N-cadherin (CDH2) given the vascular phenotype and lethality observed in the corresponding deficient mice [32], and that it mediates VSMC survival and migration, as well as pericyte recruitment by endothelial cells [33], [34]. However, although *Cdh2* also appeared downregulated in *Prkcd* and *Prkce* deficient embryos (fig. 7), immnofluorescent detection of CDH2 in mouse embryo sections showed nervous system and heart as the main sites of expression, whereas endothelium/mural cell interconnections did not show an obvious positive signal over the background *in vivo* (see Figure S1). This is in agreement with previous data regarding the expression pattern of CDH2 in mouse embryos [35]. Therefore, the downregulated transcriptional levels for *cdh2* found in double deficient embryos via qPCR is likely addressing a potential downregulation in other embryonic domains than endothelium in *Prkcd* and *Prkce* deficient embryos.

*Ets-1* belongs to the ets family, which consists of transcription factors involved in vasculo- and angiogenesis [16]. Although we found downregulated *Ets-1*, *Ets-1* null mice are viable, probably due to redundancy among ETS members [16]. Therefore, the mechanism(s) that explain embryonic lethality in the absence of PRKCD and PRKCE cannot be explained through a pathway that only involves ETS-1. We thus looked further within the same family in an attempt to find a candidate(s) whose deficiency could be consistent with the phenotype displayed by *Prkcd* and *Prkce* double deficient embryos, and were able to identify downregulated *Fli-1* (Fig. 7), an ets family member whose deficiency in mice leads to embryonic lethality due to hemorrhage and disrupted vessel integrity at approximately E12.5 [16]. However, conditional deletion of *Fli-1* in the endothelium does not prevent mouse viability, although it regulates genes involved in vascular homeostasis, such as *Cdh5* and *Cd31* [36], which are downregulated in *Prkcd* and *Prkce* double deficient embryos. This therefore opens the possibility to the existence of a mechanism involved, at least, in vascular homeostasis where *Prkcd* and *Prkce* might regulate *Cdh5* and *Cd31* via *Fli-1* and *Ets-1*, since these genes are all regulated in *Prkcd* and *Prkce* double deficient embryos (fig.7).

We also looked into mRNA levels via semiquantitative PCR for *Mef2c*, a transcription factor that contains essential ETS binding sites for vascular development and viability in mouse embryos at E9.5 [37], but did not observe any clear regulation in double deficient embryos (data not shown), which could be due to redundant function that exists within the *Ets* family [16]. The observed significant downregulation of the endothelial markers *Cd31* and *Vegfr2* (*Flk-1*) in double deficient embryos via qPCR (Fig. 7) could also be due to fewer endothelial cells present in double deficient embryos. Indeed, *Flk-1* deficient mice die at E8.5 and display impaired vasculogenesis [38], so *Flk1* seems to lie upstream *Prkcd* and *Prkce*. *Cd31* deficient mice, however, are viable and do not display a vascular phenotype [39], so PRKCD and PRKCE dependent *Cd31* expression should not be crucial in mouse viability.

Regarding ERK, a downstream target in VEGF signaling, it can be activated by PRKCD [40] and PRKCE [11]. Moreover, endothelial specific *Erk1/2* deficient mice are also embryonic lethal due to an angiogenic phenotype at approximately E9.5 [15]. For this reason we hypothesized that *Prkcd* and *Prkce* could lie upstream ERK signaling in endothelium, and screened the most significantly regulated genes in the absence of endothelial ERK [15]. We thus found downregulation of the cell division/proliferation related genes *Aurka*, *Ccne* and *Mcm2* (Fig. 7). However, the adhesion and migration mediating integrin subunit beta1 (*Itgb1*), which also appeared downregulated in *Erk*1/2 null embryos, did not appear significantly regulated in our double deficient embryos (Fig. 7). Consistently, hemidesmosomes were detectable in the absence of PRKCD and PRKCE (Fig. 5F). Thus, a more complex mechanism that explains embryonic vessel formation may exist.

In summary, our data show for the first time that PRKCD and PRKCE have a redundant role *in vivo* that is necessary for blood vessel formation during mouse embryogenesis via a mechanism that may involve several angiogenic genes, such as *Cdh2*, *Cdh5*, *Ets-1* and *Fli-1*. This vascular phenotype might be a major contributor to the observed embryonic lethality, although additional phenotypes that could also prevent further development and contribute to lethality might also exist at the same embryonic stage. Our data, however, still point at PRKCD and PRKCE as potentially good targets when considering inhibition of vasculo- and/or angiogenesis.

## Supporting Information

Figure S1 **N-cadherin (CDH2) expression at E9.5 in wild type embryos.** A, Sites with clear detection of N-cadherin were neuroepithelium and heart. Arrows indicate dorsal aorta. B, No clear signal over background for N-cadherin could be observed at interconnections (arrows) between endothelial and mural cells in dorsal aorta. Scale bars  = 100 µm (A) and 50 µm (B).
(TIF)

Table S1 **List of primers used for qPCR.**
(XLS)

## References

[1] Duquesnes N, Lezoualc'h F, Crozatier B (2011) PKC-delta and PKC-epsilon: Foes of the same family or strangers? Journal of molecular and cellular cardiology 51: 665–673. 21810427 10.1016/j.yjmcc.2011.07.013

[2] Geraldes P, Hiraoka-Yamamoto J, Matsumoto M, Clermont A, Leitges M, et al (2009) Activation of PKC-delta and SHP-1 by hyperglycemia causes vascular cell apoptosis and diabetic retinopathy. Nat Med 15: 1298–1306. 19881493 10.1038/nm.2052 PMC3290906

[3] Meier M, Menne J, Park JK, Holtz M, Gueler F, et al (2007) Deletion of protein kinase C-epsilon signaling pathway induces glomerulosclerosis and tubulointerstitial fibrosis in vivo. J Am Soc Nephrol 18: 1190–1198. 17360953 10.1681/ASN.2005070694

[4] Bekhite MM, Finkensieper A, Binas S, Muller J, Wetzker R, et al (2011) VEGF-mediated PI3K class IA and PKC signaling in cardiomyogenesis and vasculogenesis of mouse embryonic stem cells. J Cell Sci 124: 1819–1830. 21540297 10.1242/jcs.077594

[5] Monti M, Donnini S, Morbidelli L, Giachetti A, Mochly-Rosen D, et al (2013) PKCepsilon activation promotes FGF-2 exocytosis and induces endothelial cell proliferation and sprouting. J Mol Cell Cardiol 63C: 107–117. 10.1016/j.yjmcc.2013.07.006 PMC3812807 23880610

[6] Koh W, Mahan RD, Davis GE (2008) Cdc42- and Rac1-mediated endothelial lumen formation requires Pak2, Pak4 and Par3, and PKC-dependent signaling. J Cell Sci 121: 989–1001. 18319301 10.1242/jcs.020693

[7] Harrington EO, Shannon CJ, Morin N, Rowlett H, Murphy C, et al (2005) PKCdelta regulates endothelial basal barrier function through modulation of RhoA GTPase activity. Exp Cell Res 308: 407–421. 15935342 10.1016/j.yexcr.2005.05.005

[8] Bai X, Margariti A, Hu Y, Sato Y, Zeng L, et al (2010) Protein kinase C{delta} deficiency accelerates neointimal lesions of mouse injured artery involving delayed reendothelialization and vasohibin-1 accumulation. Arterioscler Thromb Vasc Biol 30: 2467–2474. 20884873 10.1161/ATVBAHA.110.215723

[9] Gorshkova I, He D, Berdyshev E, Usatuyk P, Burns M, et al (2008) Protein kinase C-epsilon regulates sphingosine 1-phosphate-mediated migration of human lung endothelial cells through activation of phospholipase D2, protein kinase C-zeta, and Rac1. J Biol Chem 283: 11794–11806. 18296444 10.1074/jbc.M800250200 PMC2431079

[10] Deuse T, Koyanagi T, Erben RG, Hua X, Velden J, et al (2010) Sustained inhibition of epsilon protein kinase C inhibits vascular restenosis after balloon injury and stenting. Circulation 122: S170–178. 20837910 10.1161/CIRCULATIONAHA.109.927640

[11] Rask-Madsen C, King GL (2008) Differential regulation of VEGF signaling by PKC-alpha and PKC-epsilon in endothelial cells. Arterioscler Thromb Vasc Biol 28: 919–924. 18323518 10.1161/ATVBAHA.108.162842 PMC3340425

[12] Lizotte F, Pare M, Denhez B, Leitges M, Guay A, et al (2013) PKCdelta Impaired Vessel Formation and Angiogenic Factor Expression in Diabetic Ischemic Limbs. Diabetes 62: 2948–2957. 23557702 10.2337/db12-1432 PMC3717846

[13] Caprioli A, Minko K, Drevon C, Eichmann A, Dieterlen-Lievre F, et al (2001) Hemangioblast commitment in the avian allantois: Cellular and molecular aspects. Developmental Biology 238: 64–78. 11783994 10.1006/dbio.2001.0362

[14] Patel-Hett S, D'Amore PA (2011) Signal transduction in vasculogenesis and developmental angiogenesis. Int J Dev Biol 55: 353–363. 21732275 10.1387/ijdb.103213sp PMC4075038

[15] Srinivasan R, Zabuawala T, Huang H, Zhang J, Gulati P, et al (2009) Erk1 and Erk2 regulate endothelial cell proliferation and migration during mouse embryonic angiogenesis. PLoS One 4: e8283. 20011539 10.1371/journal.pone.0008283 PMC2789384

[16] De Val S, Black BL (2009) Transcriptional control of endothelial cell development. Dev Cell 16: 180–195. 19217421 10.1016/j.devcel.2009.01.014 PMC2728550

[17] Hashiya N, Jo N, Aoki M, Matsumoto K, Nakamura T, et al (2004) In vivo evidence of angiogenesis induced by transcription factor Ets-1: Ets-1 is located upstream of angiogenesis cascade. Circulation 109: 3035–3041. 15173033 10.1161/01.CIR.0000130643.41587.DB

[18] Leitges M, Mayr M, Braun U, Mayr U, Li C, et al (2001) Exacerbated vein graft arteriosclerosis in protein kinase Cdelta-null mice. J Clin Invest 108: 1505–1512. 11714742 10.1172/JCI12902 PMC209416

[19] Lessmann E, Leitges M, Huber M (2006) A redundant role for PKC-epsilon in mast cell signaling and effector function. Int Immunol 18: 767–773. 16569674 10.1093/intimm/dxl012

[20] Carracedo S, Braun U, Leitges M (2013) Expression pattern of Protein Kinase C epsilon during mouse embryogenesis. BMC Dev Biol 13: 16. 23639204 10.1186/1471-213X-13-16 PMC3668281

[21] Carracedo S, Braun U, Leitges M (2013) Expression pattern of protein kinase C delta during mouse embryogenesis. BMC Dev Biol 13: 2. 23305608 10.1186/1471-213X-13-2 PMC3552935

[22] Zudaire E, Gambardella L, Kurcz C, Vermeren S (2011) A computational tool for quantitative analysis of vascular networks. PLoS One 6: e27385. 22110636 10.1371/journal.pone.0027385 PMC3217985

[23] Gambardella L, Zudaire E, Vermeren S (2012) Quantitative Analysis of Angiogenesis in the Allantois Explant Model. In: Zudaire E, Cuttitta F, editors. The Textbook of Angiogenesis and Lymphangiogenesis: Methods and Applications: Springer Netherlands. pp. 189–204.

[24] Acin-Perez R, Hoyos B, Gong J, Vinogradov V, Fischman DA, et al (2010) Regulation of intermediary metabolism by the PKCdelta signalosome in mitochondria. FASEB J 24: 5033–5042. 20798245 10.1096/fj.10-166934 PMC2992363

[25] Downs KM, Gifford S, Blahnik M, Gardner RL (1998) Vascularization in the murine allantois occurs by vasculogenesis without accompanying erythropoiesis. Development 125: 4507–4520. 9778509 10.1242/dev.125.22.4507

[26] Arora R, Papaioannou VE (2012) The murine allantois: a model system for the study of blood vessel formation. Blood 120: 2562–2572. 22855605 10.1182/blood-2012-03-390070 PMC3460680

[27] Ding R, Darland DC, Parmacek MS, D'Amore PA (2004) Endothelial-mesenchymal interactions in vitro reveal molecular mechanisms of smooth muscle/pericyte differentiation. Stem Cells Dev 13: 509–520. 15588508 10.1089/scd.2004.13.509

[28] Kuriyama M, Taniguchi T, Shirai Y, Sasaki A, Yoshimura A, et al (2004) Activation and translocation of PKCdelta is necessary for VEGF-induced ERK activation through KDR in HEK293T cells. Biochem Biophys Res Commun 325: 843–851. 15541367 10.1016/j.bbrc.2004.10.102

[29] Carracedo S, Braun U, Leitges M (2013) Expression pattern of protein kinase Cdelta during mouse embryogenesis. BMC Dev Biol 13: 2. 23305608 10.1186/1471-213X-13-2 PMC3552935

[30] Skalli O, Pelte MF, Peclet MC, Gabbiani G, Gugliotta P, et al (1989) Alpha-smooth muscle actin, a differentiation marker of smooth muscle cells, is present in microfilamentous bundles of pericytes. J Histochem Cytochem 37: 315–321. 2918221 10.1177/37.3.2918221

[31] Gory-Faure S, Prandini MH, Pointu H, Roullot V, Pignot-Paintrand I, et al (1999) Role of vascular endothelial-cadherin in vascular morphogenesis. Development 126: 2093–2102. 10207135 10.1242/dev.126.10.2093

[32] Radice GL, Rayburn H, Matsunami H, Knudsen KA, Takeichi M, et al (1997) Developmental defects in mouse embryos lacking N-cadherin. Dev Biol 181: 64–78. 9015265 10.1006/dbio.1996.8443

[33] Gaengel K, Genove G, Armulik A, Betsholtz C (2009) Endothelial-mural cell signaling in vascular development and angiogenesis. Arterioscler Thromb Vasc Biol 29: 630–638. 19164813 10.1161/ATVBAHA.107.161521

[34] Navarro P, Ruco L, Dejana E (1998) Differential localization of VE- and N-cadherins in human endothelial cells: VE-cadherin competes with N-cadherin for junctional localization. J Cell Biol 140: 1475–1484. 9508779 10.1083/jcb.140.6.1475 PMC2132661

[35] Luo Y, Kostetskii I, Radice GL (2005) N-cadherin is not essential for limb mesenchymal chondrogenesis. Dev Dyn 232: 336–344. 15614770 10.1002/dvdy.20241

[36] Asano Y, Stawski L, Hant F, Highland K, Silver R, et al (2010) Endothelial Fli1 deficiency impairs vascular homeostasis: a role in scleroderma vasculopathy. Am J Pathol 176: 1983–1998. 20228226 10.2353/ajpath.2010.090593 PMC2843486

[37] De Val S, Anderson JP, Heidt AB, Khiem D, Xu SM, et al (2004) Mef2c is activated directly by Ets transcription factors through an evolutionarily conserved endothelial cell-specific enhancer. Dev Biol 275: 424–434. 15501228 10.1016/j.ydbio.2004.08.016

[38] Shalaby F, Rossant J, Yamaguchi TP, Gertsenstein M, Wu XF, et al (1995) Failure of blood-island formation and vasculogenesis in Flk-1-deficient mice. Nature 376: 62–66. 7596435 10.1038/376062a0

[39] Duncan GS, Andrew DP, Takimoto H, Kaufman SA, Yoshida H, et al (1999) Genetic evidence for functional redundancy of Platelet/Endothelial cell adhesion molecule-1 (PECAM-1): CD31-deficient mice reveal PECAM-1-dependent and PECAM-1-independent functions. J Immunol 162: 3022–3030. 10072554

[40] Luo Y, Radice GL (2005) N-cadherin acts upstream of VE-cadherin in controlling vascular morphogenesis. J Cell Biol 169: 29–34. 15809310 10.1083/jcb.200411127 PMC2171890

