# Supplementary figures and images for: Redundant Role of Protein Kinase C Delta and Epsilon during Mouse Embryonic Development
Source: PLoS One. 2014 Aug 1;9(8):e103686. doi: 10.1371/journal.pone.0103686 (PMC4118884; doi:10.1371/journal.pone.0103686)

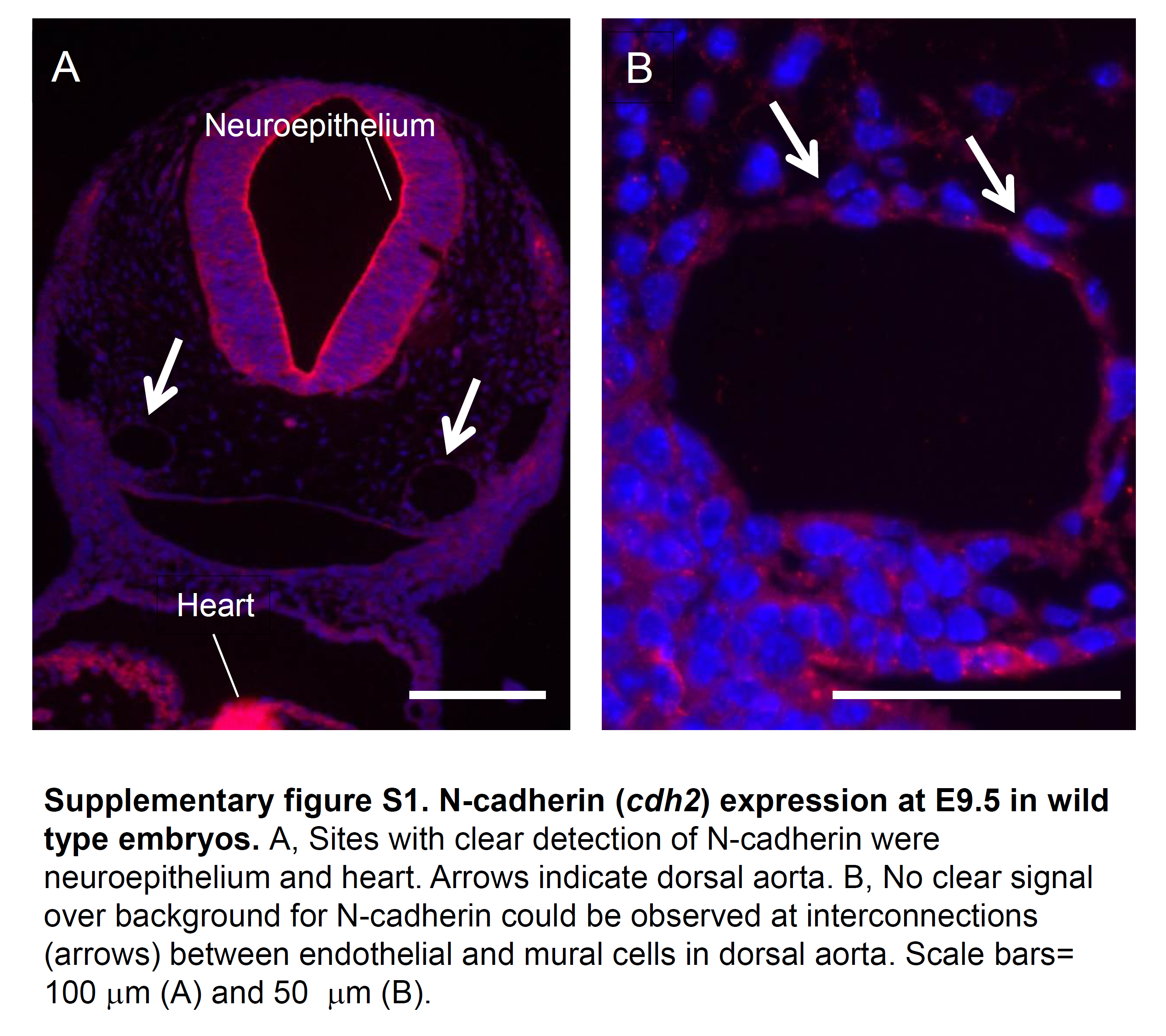

Supplement: Figure S1 — N-cadherin (CDH2) expression at E9.5 in wild type embryos. A, Sites with clear detection of N-cadherin were neuroepithelium and heart. Arrows indicate dorsal aorta. B, No clear signal over background for N-cadherin could be observed at interconnections (arrows) between endothelial and mural cells in dorsal aorta. Scale bars = 100 µm (A) and 50 µm (B). (TIF) [file pone.0103686.s001.tif]
